# Differences in medication knowledge and risk of errors between graduating nursing students and working registered nurses: comparative study

**DOI:** 10.1186/s12913-014-0580-7

**Published:** 2014-11-21

**Authors:** Bjoerg O Simonsen, Gro K Daehlin, Inger Johansson, Per G Farup

**Affiliations:** Department of Quality and Patient Safety, Innlandet Hospital Trust, Brumunddal, N-2381 Norway; Unit for Applied Clinical Research, Faculty of Medicine, Norwegian University of Science and Technology, Trondheim, Norway; Faculty of Health, Care and Nursing, Gjoevik University College, Gjoevik, Norway; Department of Nursing, Karlstad University, Karlstad, Sweden; Department of Research, Innlandet Hospital Trust, Brumunddal, Norway

**Keywords:** Nursing education, Medication, Safety, Pharmacology, Drug dose calculations, MCQ test

## Abstract

**Background:**

Nurses experience insufficient medication knowledge; particularly in drug dose calculations, but also in drug management and pharmacology. The weak knowledge could be a result of deficiencies in the basic nursing education, or lack of continuing maintenance training during working years. The aim of this study was to compare the medication knowledge, certainty and risk of error between graduating bachelor students in nursing and experienced registered nurses.

**Methods:**

Bachelor students in closing term and registered nurses with at least one year job experience underwent a multiple choice test in pharmacology, drug management and drug dose calculations: 3x14 questions with 3–4 alternative answers (score 0–42). Certainty of each answer was recorded with score 0–3, 0–1 indicating need for assistance. Risk of error was scored 1–3, where 3 expressed high risk: being certain that a wrong answer was correct. The results are presented as mean and (SD).

**Results:**

Participants were 243 graduating students (including 29 men), aged 28.2 (7.6) years, and 203 registered nurses (including 16 men), aged 42.0 (9.3) years and with a working experience of 12.4 years (9.2). The knowledge among the nurses was found to be superior to that of the students: 68.9%(8.0) and 61.5%(7.8) correct answers, respectively, (p < 0.001). The difference was largest in drug management and dose calculations. The improvement occurred during the first working year. The nurses expressed higher degree of certainty and the risk of error was lower, both overall and for each topic (p < 0.01). Low risk of error was associated with high knowledge and high sense of coping (p < 0.001).

**Conclusions:**

The medication knowledge among experienced nurses was superior to bachelor students in nursing, but nevertheless insufficient. As much as 25% of the answers to the drug management questions would lead to high risk of error. More emphasis should be put into the basic nursing education and in the introduction to medication procedures in clinical practice to improve the nurses’ medication knowledge and reduce the risk of error.

## Background

Adverse events frequently involve medication errors, and they accounted for 19% of the events reported in Norway in 2013 [1]. The events are often categorized as prescription errors made by physicians or management errors made by nurses. A common notion has been that the nurses’ involvement in medication management is quite simple: giving the Right patient the Right drug in the Right dose and Right administration form at the Right time. However, the nurses’ responsibilities include more than just carrying out the orders from the physicians [2]. In order to ensure a safe storage, dispensing and administration of the drugs, the nurses must know the pharmacological principles for each drug; the Regulations of drug management; precautions for preparation; and considerations concerning administration to patients. Errors may be caused by either lack of knowledge, routine failure, insufficient practical skills or as a result of an accidental happening [3].

During the years there has been considerable concern about the insufficient drug dose calculation skills among nursing students and registered nurses [4-8]. Questions have also been raised about the basic pharmacology education in university colleges, but this has not been studied to the same extent [9,10]. Little is known about the scope of knowledge in drug management. A german observation study investigated errors during the drug management process, and found errors in 61% of the observed cases: storage errors in 27%, dispensing errors in 88% and administration errors in 36% [11].

The students are under supervision and not allowed to perform medication tasks by themselves, but there have been documented administration errors in 26–40% of the processes [12]. In a study analysing more than 1300 student medication errors in the USA, 51% was caused by performance deficits and 27% by knowledge deficit [13]. Other studies around the world confirm that the issue of medication errors among nursing students should be taken seriously [14,15].

It seems predictable that experienced nurses have better knowledge and practical skills than graduating nurses. In an earlier study, we concluded that medication knowledge was unsatisfactory among practicing nurses, with a significant risk for medication errors [16]. The study revealed a need to improve the nurses’ basic knowledge, especially in drug management. The insufficient medication knowledge could be a result of deficiencies in the basic nursing education, or lack of continuing maintenance training during working years. We would like to investigate this further by comparing the medication knowledge in registered nurses with the knowledge of graduating bachelor students in nursing, at closing term of the 3^rd^ year.

### Aims

The primary aim of the study was to compare medication knowledge, certainty and risk of error between graduating bachelor students in nursing and working registered nurses.

Secondary aims were to search for factors associated with high medication knowledge and risk of error, and to evaluate how much the medication knowledge and skills among nurses are developed during postgraduate on-the-job training and experience.

## Methods

### Study design and setting

The study was designed as a comparison of two cross-sectional studies, with graduating nursing students and registered nurses as the target groups. The participants completed the same form with relevant background information and answered a multiple-choice questionnaire (MCQ) test in medication knowledge and skills; pharmacology, drug management, and drug dose calculations. An English translation of the questionnaire has previously been published in BMC Health Services Research, as an appendix to the study of medication knowledge, certainty, and risk of errors in health care [16]. The participants carried out a test under controlled conditions, and the maximum time allowed was 2.5 h.

### Participants

Norwegian bachelor students in closing term of the 3^rd^ year and registered nurses with at least one year job experience from hospital or primary health care establishments were invited to carry out a multiple choice test in pharmacology, drug management and drug dose calculations. The students were recruited from two University colleges, with 520 3^rd-^year students, and the registered nurses were recruited from two Norwegian hospitals with 2.300 nurses and from three municipalities with 500 nurses.

Inclusion criteria were registered nurses with at least one year of work experience in 50% part time job or more. Nurses working in outpatient clinics were excluded, together with any who did not administer drugs, or who were not sufficiently fluent in Norwegian language. The study was performed from September 2007 to April 2009.

### Variables

The following demographic characteristics were recorded: age, gender, and childhood in or outside Norway. Some educational and working background information were recorded: number of years of studying mathematics beyond the first mandatory year at upper secondary school; other education prior to nursing; and percentage part time job (1 = full-time) for the past 12 months. Further, the frequency of the following medication tasks were recorded, scored 0–3: 0 = less than monthly, 1 = monthly, 2 = weekly, and 3 = every working day: calculation of dosages; preparation tasks (preparation of infusions or injections, multi dose packaging, and preparation from original package); and distribution tasks (giving to patient injections or infusions, administration from multi dose packages or single dosages). In addition, statements regarding sense of coping and self-esteem and wellbeing related to medication tasks were recorded, using parts of the General Health Questionnaire (GHQ 30), a Quality of life tool on psychological and psychosocial symptoms [17].

The medication knowledge test consisted of 42 multiple-choice questions with 3–4 alternative answers. The topics within the disciplines pharmacology, drug management and drug dose calculations, were as follows (number of questions for each topic shown in brackets):

*Pharmacology (14)*: general pharmacology (3), effect (3), side effects and interactions (4), administration form and generics (4).

*Drug management (14):* regulations (2), storage (4), dispensing (4), and distribution (4).

*Drug dose calculations (14)*: conversion of units (7), formulas for calculation of dose, quantity or strength (4), and infusion and dilution (3).

The questions were put together from actual tests for bachelor nursing students at university colleges, from tests of continuing educational programs used in Norwegian hospitals, and some were guided from experience from problems arising among nurses. The questionnaire was tested for comprehension by 5 experienced registered nurses working in nursing home, hospital wards and intensive care units. The quality assurance of face validity did not result in need for revisions of the questionnaire. The results are presented as per cent correct answers.

For each question the participants indicated a self-estimated certainty, graded from 0–3: 0 = very uncertain (would seek for help, consulted colleagues or reference books), 1 = relatively uncertain (would probably seek for help), 2 = relatively certain (would probably not seek for help), and 3 = very certain (would not seek for help). The results are presented as mean score.

Risk of error was defined as a combination of knowledge and certainty for each question, rated on a scale from 1 to 3 devised for the study. A correct answer combined with high certainty (relatively or very certain) was stated as low risk of error (score = 1), any answer combined with low certainty (relatively or very uncertain) was stated as moderate risk of error (score = 2), and incorrect answer combined with high certainty (relatively or very certain) was regarded as high risk of error (score = 3). The results are presented as mean score, or as percent answers with high risk of error.

Nine statements from the GHQ30 questionnaire were answered: Five statements about coping (finding life a struggle; being able to enjoy normal activities; feeling reasonably happy; getting scared or panicky for no good reason; and being capable of making decisions), and four statements about self-esteem and wellbeing (overall doing things well; satisfied with the way they have carried out their task; managing to keep busy and occupied; and managing as well as most people in the same situation). The statements were scored 0–3: 0 = more or better than usual, 1 = as usual, 2 = less or worse than usual and 3 = much less or worse than usual; “as usual” was defined as the normal state.

### Ethics

The Privacy Ombudsman for Research at a regional university Hospital, representing The Norwegian Data Inspectorate, approved the study. Further approval from Regional Committees for Medical and Health Research Ethics was not demanded. Participation was voluntarily, and all participants gave written informed consent before inclusion. The data were collected de-identified, and all data were made anonymous before analyses, to protect the participants from any consequences as a result of the test. It was considered ethically justifiable that it would not be possible to identify persons with high risk of error from this study. Risky behavior should be caught up by an internal quality management system.

### Analyses

#### Power calculation

In a former cross-sectional study, 203 registered nurses were tested for medication knowledge [16]. With 200 participants there was a power of 0.9 to detect a difference of 1 point out of 14 between two groups, with p < 0.05, provided 75% right answers (score 10.5) and 15% SD (2.1). Earlier studies testing drug dose calculation skills among nurses was used as a reference for the knowledge level [4,5,18]. The students were recruited to get a convenient group for comparison.

#### Statistical methods

Depending on data distribution, comparisons between groups were analyzed with Chi-square or Fishers exact test; t-test or Mann-Whitney U-test; ANOVA or Kruskal-Wallis; and Pearson or Spearman tests for correlations. Friedman’s test was used for comparisons between the disciplines measured by the same scale. Independent factors associated with high knowledge and high risk of error were analyzed by standard linear regression after checking for multicolinearity and residual normality [19]. Two-tailed significance tests were used, and p-value <0.05 was considered statistically significant. The study protocol predefined how to handle missing data. Unanswered questions or statements were scored as “incorrect answer”, and unanswered certainty was scored as “very uncertain”. The analyses were performed with SPSS version 18.0 (SPSS Inc., Chicago, IL, USA).

## Results

**Figure 1:**
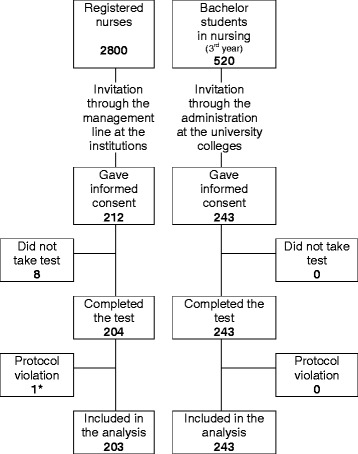
**Flow diagram of the participants.** ^*)^Nurse was not working at the time of inclusion.

In total, 243 students and 212 registered nurses were included in the study. Figure 1 shows a flow diagram of the participants, and Table 1 summarizes the background characteristics. The mean working experience among the nurses were 12.4 years (SD 9.2), ranging from 1–42 years. They were equally recruited from hospitals (99 nurses) and primary health care establishments (104 nurses), 68 of them (33.5%) had taken postgraduate specialization, and 46 nurses (22.6%) had participated in relevant medication courses during the past 3 years. Median frequency of medication management tasks performed by the nurses were: every day for preparation of single doses and distribution from multi-dose packages; weekly for dose calculations, preparation of multi-dose packages, and distribution of single doses and infusions or injections to patient; and less than weekly for preparation of infusions or injections.

**Table 1:** **Characteristics of participants**

	**Students**	**Nurses**	**P-value**
	**(n = 243)**	**(n = 203)**	
Age in years (SD), range (n = 441)^1)^	28.2 (7.6), 21–54	42.0 (9.3), 23–66	<0.001
Gender (men)	29 (11.9%)	16 (7.9%)	0.16
Childhood outside Norway (n = 445)^1)^	12 (5.0%)	16 (7.9%)	0.21
Mathematics beyond 1^st^ year high-school/USS^2)^	105 (43.2%)	80 (39.4%)	0.42
Other education prior to nurse education	106 (43.6%)	81 (39.9%)	0.43
Part time job past 12 months (full time = 1.00)	0.23 (0.21)	0.86 (0.16)	<0.001
**Frequency** ^**3)**^ **medication tasks - overall**	0.6 (0.5)	1.7 (0.6)	<0.001
- Drug dose calculation tasks	0.2 (0.5)	1.4 (1.1)	<0.001
- Preparation tasks	0.3 (0.5)	1.8 (0.6)	<0.001
- Distribution tasks	0.9 (0.8)	1.8 (0.7)	<0.001
**Total mean score GHQ** ^**4)**^ (9 items)	0.89 (0.26)	0.90 (0.23)	0.67
- Sense of coping (0–3)	0.97 (0.27)	0.79 (0.28)	<0.001
- Sense of self esteem/well-being (0–3)	0.79 (0.35)	1.01 (0.23)	<0.001

The results are given as mean (standard deviation), or number of participants (proportion).

^1)^Variable with missing data.

^2)^Upper secondary school.

^3)^Frequency: 0 = less than monthly, 1 = monthly, 2 = Weekly, 3 = every working day.

^4)^General Health Questionnaire (GHQ) score 0–3, 0 = better than usual, 1 = as usual, 2 = worse than usual, 3 = much worse than usual.

The two groups were well balanced in terms of demography and other characteristics. The students were significantly younger, had a smaller part time job than the registered nurses, and they performed medication tasks more seldom. The nurses expressed a better sense of coping in medication tasks while the students indicated better self-esteem and wellbeing.

Table 2 summarizes the primary outcomes of the MCQ test in medication knowledge, certainty evaluation and risk of error, totally and for each discipline and topic. The nurses scored statistically higher than the students in knowledge and certainty assessment, both overall and for each discipline, and lower in the corresponding risks of error, with the exception of the topics general pharmacology and dispensing tasks. Students scored higher than nurses in dispensing knowledge and had a lower risk of error in this discipline than nurses.

**Table 2:** **Primary outcomes of the MCQ test in medication knowledge, certainty evaluation and risk of error – totally, for each discipline and topic**

	**Knowledge**		**Certainty in each answer**		**Risk of error**	
	**Proportion correct answers**		**Score 0**–**3** ^**1)**^		**Score 1**–**3** ^**2)**^	
	**Students**	**Nurses**	**Students**	**Nurses**	**Students**	**Nurses**
**Total**	**61.5 (7.8)**	**68.9 (8.0)**	**1.7 (0.4)**	**1.9 (0.4)**	**1.8 (0.1)**	**1.7 (0.1)**
**Pharmacology (14)** ^**3)**^	**70.8 (11.4)**	**73.7 (11.1)**	**1.6 (0.5)**	**1.8 (0.5)**	**1.7 (0.2)**	**1.6 (0.2)**
- General (3)	84.5 (19.7)	81.9 (21.8)	1.8 (0.6)	1.70.6)	1.5 (0.3)	1.5 (0.4)
- Effect (3)	72.8 (25.0)	79.8 (20.8)	1.3 (0.7)	1.6 (0.7)	1.6 (0.3)	1.5 (0.3)
- Side effects, interactions (4)	71.3 (22.7)	77.1 (21.8)	1.5 (0.6)	1.8 (0.7)	1.7 (0.3)	1.6 (0.3)
- Administration form/generics (4)	58.5 (20.6)	59.5 (19.1)	1.8 (0.5)	1.9 (0.6)	1.9 (0.3)	1.8 (0.3)
**Drug management (14)**	**42.7 (11.8)**	**53.2 (11.8)**	**1.6 (0.5)**	**1.9 (0.5)**	**2.0 (0.2)**	**1.9 (0.2)**
- Responsibility (2)	27.4 (33.7)	58.9 (34.4)	1.5 (0.8)	2.0 (0.7)	2.2 (0.5)	1.8 (0.6)
- Storage (4)	17.3 (18.0)	33.9 (21.4)	1.2 (0.7)	1.5 (0.6)	2.3 (0.3)	2.1 (0.4)
- Dispensing (4)	61.6 (19.7)	60.1 (21.5)	1.7 (0.6)	2.0 (0.6)	1.7 (0.3)	1.8 (0.4)
- Distribution (4)	56.9 (22.6)	62.8 (22.2)	1.8 (0.7)	2.1 (0.6)	2.0 (0.4)	1.8 (0.4)
**Drug dose calculations (14)**	**71.0 (13.5)**	**79.9 (14.2)**	**1.8 (0.5)**	**2.0 (0.6)**	**1.6 (0.2)**	**1.5 (0.3)**
- Units (7)	71.4 (17.7)	75.7 (20.4)	1.9 (0.5)	2.1 (0.6)	1.6 (0.2)	1.5 (0.3)
- Dose-amount-strength (4)	71.5 (19.1)	84.7 (16.9)	1.9 (0.6)	2.0 (0.7)	1.5 (0.3)	1.4 (0.3)
- Dilution/infusion (3)	67.7 (25.9)	83.4 (25.3)	1.5 (0.8)	1.8 (0.8)	1.6 (0.4)	1.5 (0.4)

The results are given as percent or mean score, both with (SD). Mann–Whitney U-test.

^1)^Scale: 0 = very uncertain, 1 = relatively uncertain, 2 = relatively certain, 3 = very certain.

^2)^Scale: 1 = low risk, 2 = moderate risk, 3 = high risk.

^3)^( ) = Number of questions.

Comparisons between the 3 disciplines for proportion of correct answers, mean score of certainty and mean score of risk of error (Friedman test) were statistically significant; p-values ≤0.001.

There was no difference between the study groups in overall risk of error. More detailed information is given in Table 3. The only discipline where nurses were significantly safer than the students was drug dose calculations, with fewer high-risk answers in the dose-amount-strength calculations. In drug management, 26,6% and 26,4% of the answers from students and nurses, respectively, led to a high risk of error. The contributing factors to the differences between the groups in high risk of error in the drug management topics are also found in Table 2: The students revealed a lower knowledge of regulations, and the nurses stated a higher degree of certainty in the dispensing questions.

**Table 3:** **High risk of error estimated from medication knowledge and certainty evaluation - totally, for each discipline and topic**

	**Proportion of questions with high risk of error**		
	**Students**	**Nurses**	**P-value**
	**(n = 243)**	**(n = 203)**	
**Total (42)** ^**2)**^	**15.6%**	**14.9%**	**0.46**
**Pharmacology (14)**	11.2%	11.1%	0.98
- General (3)	5.9%	5.4%	0.68
- Effect (3)	3.7%	2.8%	0.38
- Side effects/interactions (4)	7.9%	8.9%	0.66
- Administration form/generics (4)	24.0%	23.8%	0.99
**Drug management**	**26.6%**	**26.4%**	**0.87**
- Responsibility (2)	36.4%	24.9%	0.001
- Storage (4)	29.8%	27.3%	0.39
- Dispensing (4)	15.0%	24.3%	<0.001
- Distribution (4)	30.0%	28.4%	0.43
**Drug dose calculations (14)**	**9.1%**	**7.2%**	**0.005**
- Units (7)	10.8%	10.2%	0.30
- Dose-amount-strength (4)	8.4%	3.4%	<0.001
- Dilution/infusion (3)	5.5%	4.8%	0.38

Mann–Whitney U-test.

The results are given as percent of the total number of answers where the participants were certain that an incorrect answer was correct.

^2)^( ) = number of questions.

**Figure 2:**
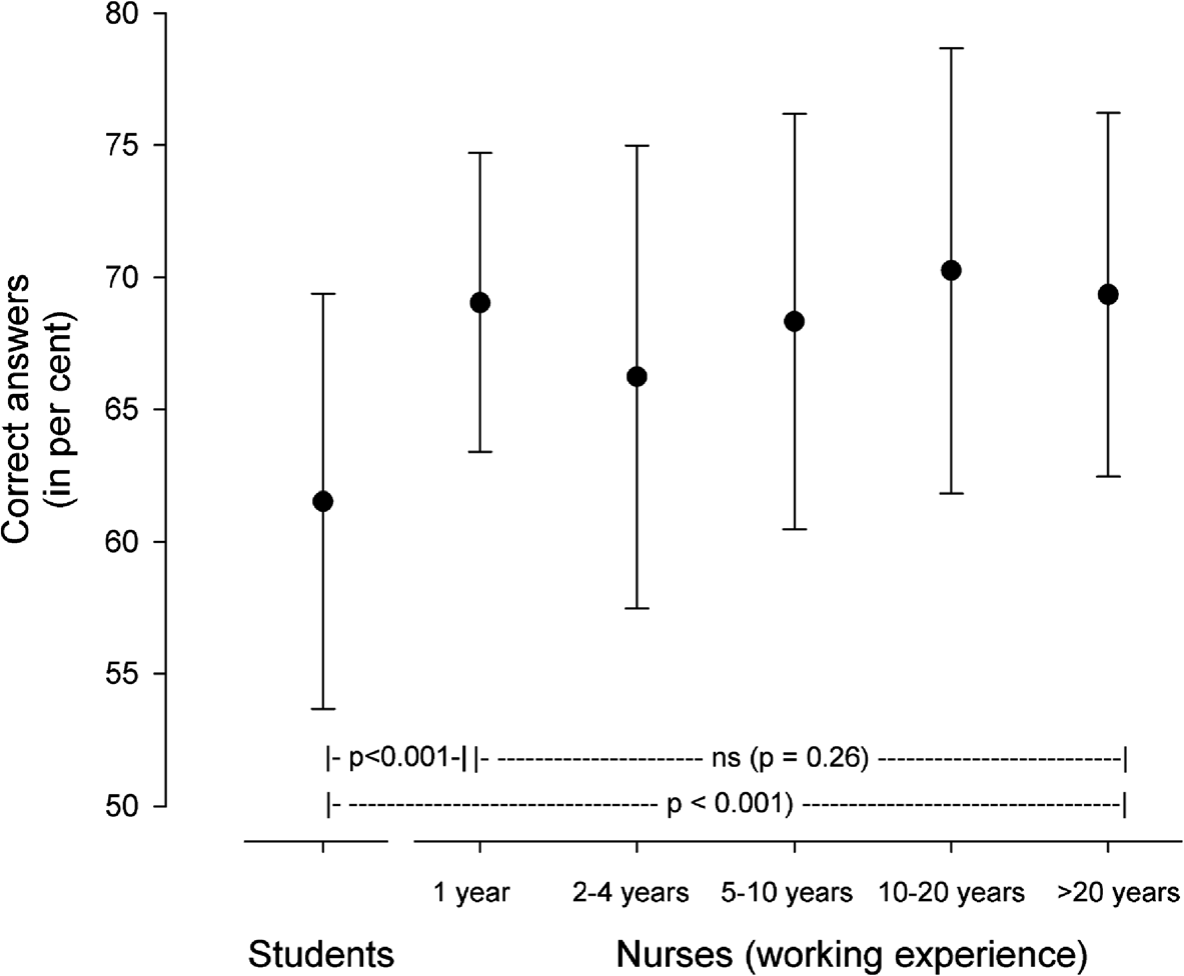
**Overall medication knowledge in the MCQ test with increasing working experience.** The results are given as mean and SD.

Figure 2 shows the results of the medication knowledge test with the number of years of working experience. A significant increase in knowledge was identified after one year, but no further increase was demonstrated.

Factors associated with high medication knowledge are given in Table 4.

**Table 4:** **Knowledge - association between high medication knowledge, totally and for each discipline, and the participants’ background characteristics**

	**Total**		**Pharmacology**		**Drug management**		**Drug dose calculations**	
	**B (95% CI)**	**P-value**	**B (95% CI)**	**P-value**	**B (95% CI)**	**P-value**	**B (95% CI)**	**P-value**
Study group (student – > nurse)	8.05 (5.86:10.24)	<0.001	5.32 (2.22:6.41)	0.001	10.87 (7.32:14.42)	<0.001	5.87 (1.58:10.16)	0.007
Age (years)	−0.02 (−0.11:0.07)	0.69	−0.09 (−0.22:0.03)	0.15	0.08 (−0.05:0.22)	0.22	−0.04 (−0.20:0.11)	0.59
Gender (men- > women)	- 2.76 (−5.25:-0.28)	0.03	−0.57 (−4.08:2.95)	0.75	−1.68 (−.39:2.04)	0.38	−5.79 (−10.06:-1.51)	0.008
Childhood outside Norway	−3.53 (−6.64:-0.42)	0.03	−5.39 (9.79:-0.98)	0.02	−1.40 (−6.05:3.24)	0.55	−4.86 (−10.24:0.53)	0.08
Maths beyond 1 year USS^1)^	0.48 (−1.04:2.01)	0.53	−1.40 (−3.56:0.76)	0.43	0.80 (−0.48:3.08)	0.49	2.00(−0.61:4.62)	0.13
Other education prior to nurse	−0.10 (−1.64:1.43)	0.90	0.88 (−1.30:3.05)	0.20	0.50 (−1.80:2.80)	0.67	−1.88(−4.52:0.77)	0.16
Part time job	*)		*)		*)		*)	
Sense of coping^2)^	- 0.16 (−3.18:2.86)	0.92	2.84 (−1.43:7.11)	0.19	0.09 (−4.43:4.60)	0.97	−3.13 (−8.32:2.05)	0.24
Sense of wellbeing/self esteem^2)^	−0.82 (−3.61:1.96)	0.56	−2.24 (−6.18:1.71)	0.27	−1.13 (−5.29:3.04)	0.60	1.11(−3.67:5.89)	0.65
Frequency of medication tasks^3)^	*)		*)					
Dispensing					*)			
Distribution					−1.30(−2.79:0.18)	0.09	-	-
Drug dose calculations					-	-	2.59 (1.07:4.11)	0.001
R-square change	0.20		0.05		0.18		0.15	

Standard linear regression.

*)Omitted; correlation with study group >0.7 [19].

^1)^Upper secondary school.

^2)^General Health Questionnaire (GHQ) score 0–3, 0 = better than usual, 1 = as usual, 2 = worse than usual, 3 = much worse than usual.

^3)^Frequency: 0 = less than monthly, 1 = monthly, 2 = Weekly, 3 = every working day.

Study group was the primary factor associated with high medication knowledge, both overall and for each discipline. Men’s superiority in drug dose calculations contributes to significant higher medication knowledge, and a lower score for participants from outside Norway in pharmacology explains the lower overall knowledge. Frequency of drug dose calculations was positively associated with high performance in the calculation part of the test.

However, the study group was no longer associated with risk of error when limited to the defined high risk, as shown in Table 5. The predominant factors for predicting low risk of error was high knowledge and high sense of coping mainly in drug management. Given the same knowledge, childhood outside Norway was associated with a higher risk in pharmacology and drug management, and more frequent drug dose calculations was associated with a higher risk of error in this discipline.

**Table 5:** **High risk of error - association between the number of incorrect answers which the participants were certain were correct, totally and for each discipline, and the participants’ background characteristics and knowledge**

	**Total**		**Pharmacology**		**Drug management**		**Drug dose calculations**	
	**B (95% CI)**	**P-value**	**B (95% CI)**	**P-value**	**B (95% CI)**	**P-value**	**B (95% CI)**	**P-value**
Study group (student - > nurse)	0.80 (−0.19: 1.79)	0.11	0.14 (−0.17: 0.46)	0.37	0.28 (−0.40: 0.97)	0.41	0.07 (−0.32: 0.47)	0.71
Age (years)	0.005 (−0.03: 0.04)	0.79	<−0.01(−0.02:0.01)	0.42	0.01 (−0.01: 0.04)	0.34	<0.01 (−0.01: 0.02)	0.44
Gender (men- > women)	−0.99 (−2.06: 0.08)	0.07	−0.35 (−0.71:0.001)	0.05	−0.42 (−1.11: 0.27)	0.23	−0.12 (−0.51: 0.27)	0.55
Childhood outside Norway	1.87 (0.53: 3.20)	0.006	0.46 ( 0.01: 0.90)	0.04	1.11 ( 0.26: 1.96)	0.01	0.33 (−0.16: 0.82)	0.18
Maths beyond 1 year USS^1)^	−0.47 (−1.12: 0.19)	0.16	−0.19 (−0.41: 0.03)	0.09	−0.24 (−0.66: 0.18)	0.26	−0.03 (−0.26: 0.21)	0.83
Other education prior to nurse	0.31 (−0.35:0.97)	0.35	0.15 (−0.07: 0.37)	0.19	<0.01 (−0.43:0.42)	0.98	0.16 (−0.08: 0.41)	0.18
Part time job	*)		*)		*)		*)	
Sense of coping^3)^	−1.66 (−2.98:-0.33)	0.01	−0.36 (−0.80: 0.08)	0.11	−1.14 (−1.99:-0.29)	0.01	−0.07 (−0.55: 0.42)	0.78
Sense of wellbeing/self esteem^2)^	0.59 (−0.61: 1.80)	0.33	0.23 (−0.17: 0.63)	0.25	0.32 (−0.46: 0.10)	0.42	0.01 (−0.45: 0.43)	0.96
Knowledge – total	−0.15 (−0.19:-0.11)	<0.001	-		-	-	-	-
Pharmacology	-		−0.03 (−0.04:-0.02)	<0.001	-	-	-	-
Drug management	-		-		−0.07 (−0.09:-0.06)	<0.001	-	-
Drug dose calculations	-		-		-	-	−0.03 (−0.04:-0.03)	<0.001
Frequency of medication tasks^3)^	*)		*)		-	-	-	-
Dispensing					*)		-	-
Distribution					0.12 (−0.16: 0.39)	0.41	-	-
Drug dose calculations					-	-	0.15 (0.02:0.29)	0.03
R square change	0.15		0.13		0.17		0.14	

Standard linear regression.

*)Omitted; correlation with study group >0.7 [19].

^1)^Upper secondary school.

^2)^General Health Questionnaire (GHQ) score 0–3, 0 = better than usual, 1 = as usual, 2 = worse than usual, 3 = much worse than usual.

^3)^Frequency: 0 = less than monthly, 1 = monthly, 2 = Weekly, 3 = every working day.

## Discussion

The registered nurses showed significantly better medication knowledge than the bachelor students in their last term before graduation. However, our earlier research concluded that medication knowledge was unsatisfactory among the group of practising nurses, with a significant risk for medication errors [16].

Although all disciplines were statistically significant in favor of the nurses, it was considered that the difference should be at least 1 correct answer of the 14 to be meaningful clinically. Such difference was detected in drug management and drug dose calculations, but not in pharmacology, the most theoretical discipline. The knowledge profile was the same in both groups, and, somewhat surprising, drug management was the weakest discipline.

### Pharmacology

A basic knowledge of pharmacological principles is required to make proper drug management decisions and to be able to educate patients about their medication [20]. Other studies confirm an insufficient knowledge among nurses in pharmacology, and the need for more targeted education [10,16,20-23]. In our study, both nurses and students scored low in the topic administration forms and generics, and 1 out of 4 revealed a high risk of error. This finding is alarming, since the nurses have an independent responsibility for distributing the drugs, and to teach the patients how to use their medication right.

It is common that nurses teach pharmacology in the bachelor nursing studies. It has been perceived too advanced when physicians or pharmacists have been teaching. In an English survey in 52 university nursing departments, it was reported that the lecturers got no training in the teaching of pharmacology at 88% of the institutions [24]. Pharmacists should be involved in the planning of the pharmacology teaching, especially in pharmacokinetics and drug formulations.

The time allocated for basic teaching in pharmacology seems not to be in accordance with the time that nurses spend with medication tasks, which is stated up to 40% of the time [25]. Both teachers and students agree on the need for addressing pharmacological issues throughout the study, not just the first year.

Insufficient understanding of the basic pharmacology may have further implications. Advanced pharmacology is essential for the understanding of the treatment of severely ill patients. During the postgraduate nursing specialization, the basic principles are assumed known, and knowledge deficiencies could lead to a higher risk for the most vulnerable patients [26]. This result underlines the importance of pharmacology as a core subject for continuously teaching and training during the bachelor study in nursing.

### Drug management

The participants showed the weakest knowledge and highest mean risk of error in the discipline of drug management. This result was considered worrying because the nurses frequently have full responsibility for the storage, preparation and distribution tasks, and more than 25% of the answers implied a high risk of error. This was quite similar to what others have found. In a german study testing a classification model for preventing medication handling errors, 30.9% of the observed errors by experienced nurses were classified as high risk, and in a french study they found a 15% error rate in medication administration, and about 1/3^rd^ were classified as high risk [11,27].

Insufficient knowledge in drug management does not automatically lead to patient harm; it depends on the actual drug and the patient. The medication tasks are tightly integrated with other nursing tasks and mutually affected by these. A study of risk areas for managing IV drugs concluded that approximately 1/3 of the high risk errors were knowledge-based errors: hygiene; infusion time; incompatibilities; visual inspection; prescribing information; shelf life; labeling; and solution for infusion [11]. Besides the lack of knowledge that was demonstrated in the present study, other factors which may cause errors are routine failure, insufficient practical skills, or an accidental happening [3].

A consequence of the finding of insufficient drug management knowledge and high risk of error should be a more thorough cooperation between the teaching and training at the university colleges and the practice field. Inclusion of problem-based learning and high fidelity simulation training may be a contribution to improving the students’ ability to understand what it takes to fulfil the “five Rights” of medication management [15,28]. A lack of preparation in critical thinking is also stated as a challenge by new graduate nurses from a study of the development of professional self-concept in USA [29].

### Drug dose calculations

Many nursing students have negative experiences with a drug dose calculation test early in the study. A positive finding was, therefore, that the knowledge among the registered nurses was significantly better than among the students and the proportion with high risk of error significantly smaller. A reason for concern, however, was that conversions of units was the weakest topic among the nurses, which illustrate that there is a need to improve the conceptual understanding of the dose unit expressions. Drug dose calculation problems have been debated for years and investigated by many researchers [30-35]. The reason for this is probably due to the direct implication for patient harm [36]. An incorrect calculation means giving a wrong dose, and it can be random how potent the drug is. In several countries, students must complete a test in drug dose calculations before getting their graduation. In Norway, the test must be flawless and passed before the nurses’ first practice period. Wright has shown that students who receive repeated teaching throughout the study has shown better results than those who have not [37]. We have tested the students during their last term, and have thus been able to show what is maintained of the knowledge two years after the mandatory calculation test.

### Risk of error

We have introduced a new measure of risk of error. A lower risk of error among the nurses was a result of a combination of knowledge and certainty scores. It is difficult to transfer such a computed risk of error to clinical practice. We regarded a high risk situation in real life when the participants answered a question incorrectly and were certain it was correct, as the answer “very certain” or “relatively certain” implied that the respondent would not consult others. The nurses stated higher certainty in their answers, and this corresponded closely with the result that they also expressed a higher degree of coping in medication management tasks.

In a review article by Killam et al., knowledge and skill incompetence was highlighted as one of the characteristics of students at risk for unsafe nursing [38]. Another risk factor was overconfidence, which may correspond to our finding that the students expressed better self-esteem and well-being than the nurses.

The nurses’ higher medication knowledge seemed to have developed during the first year of practice, with no further improvement. The “reality shock” is often used as a term for the transition from supervised student to an assumingly independent registered nurse. An Australian study by Newton and McKenna describes the development of the nurses’ knowledge and skills during the first year after graduation [39]. From “gliding through” during the studies, they go through four stages when they meet reality of working life: “surviving”, “beginning to understand”, “sheltering under the umbrella” where they gain more confidence, to “knowing how to” after 11–12 months. This supports the development also seen in our study.

### Participant characteristics

The differences between the two groups in age, percentage part time job and frequency of medication tasks were as expected. Somewhat surprising was that the nurses on average performed management tasks less than every week. One explanation may be the organising of nurses in working teams, with the allocation of different tasks within the team. Another explanation is the use of multi-dose packages, prepared for either one or two weeks at the time, either by a pharmacy or at the ward. In community health care, auxiliary nurses or assistants with specialized training and certification administer prepared drugs to the patients. Students are not allowed to carry out medication management tasks on their own, but gets progressively responsible for the distribution of medicines in line with what assistants do after specific training.

Regarding the differences in assessment of coping and wellbeing and self-esteem, it seems reasonable that nurses indicate a higher degree of coping associated with medication tasks since the students still carries out these tasks under supervision. Likewise, a higher assessment of selfesteem and well-being among the students may be because they do not experience the same responsibility at work as the nurses.

In the regression analysis, growing up outside Norway was associated with lower knowledge and higher risk of errors. This result must be interpreted with some caution as childhood was not defined by age or length of stay in Norway. Any differences in background culture or language may have raised misunderstandings about the test questions themselves, but it could not be ruled out that language misinterpretations also may be the case in clinical practice.

Students follow the most experienced nurses in placement periods at hospitals. They are not allowed to take responsibility for the medication tasks, and are dependent of instructions. Students are fully aware of their role and need for supervision. Lack of basic knowledge among nurses may thus be transferred to the students [40].

### Limitations

The participants constituted an unequal proportion of the total population of students and nurses: 47% of the 3^rd-^year student population and 8% of the total nursing population was included in the study. This may have an impact on the representativeness or external validity of the results. Based on an assumption that nurses with weak knowledge hesitate to register for such a study, it is likely that the results of the student group is more representative than of the nurse group, and that the difference between students and nurses may be even less than the study shows.

Another limitation could be the questionnaire itself, which was not a validated instrument, but developed for the study. The topics and questions were selected from the essential curriculum in the nurse education framework, and were in accordance with what other investigators have described in similar tests that they have constructed [21,41]. Since our test results are consistent with what others have found, with the same conclusion that there is a need to improve the knowledge in pharmacology and drug management, this limitation is probably not crucial [20].

The study was performed some years ago. However, there has been no change in the legislation of the National curriculum for nursing education or the post-registration training [42].

## Conclusions

This study demonstrated that the medication knowledge among practising nurses was superior to that of the graduating nursing students, and the risk of error was lower. Nevertheless, even experienced nurses have insufficient skills to ensure safe medication for the patients. The results indicate that the nurses develop their medication knowledge only during the first year of practice. This study suggest a need to put more emphasis on medication in the bachelor nursing study, and to strengthen the on-the-job-training in medication procedures and pharmacological aspects beyond the first year of nursing practice.

## References

[1] Saastad E, Flesland O, Lindahl AK: ***Report 2013-The Reporting system for Adverse Events in Specialized Health Services.*** Oslo: The Norwegian Knowledge Centre for the Health Services; 2014. ISBN 978-82-8121-878-9.

[2] Jennings BM, Sandelowski M, Mark B (2011). The nurse’s medication day. Qual Health Res.

[3] Ferner RE, Aronson JK (2006). Clarification of terminology in medication errors: definitions and classification. Drug Saf.

[4] Ashby DA (1997). Medication calculation skills of the medical-surgical nurse. Medsurg Nurs.

[5] Bindler R, Bayne T (1991). Medication calculation ability of registered nurses. Image J Nurs Sch.

[6] Blais K, Bath JB (1992). Drug calculation errors of baccalaureate nursing students. Nurse Educ.

[7] Cartwright M (1996). Numeracy needs of the beginning registered nurse. Nurse Educ Today.

[8] McMullan M, Jones R, Lea S (2010). Patient safety: numerical skills and drug calculation abilities in nursing students and registered nurses. J Adv Nurs.

[9] Dilles T, Vander Stichele RR, Van Bortel L, Elseviers MM (2011). Nursing students’ pharmacological knowledge and calculation skills: ready for practice?. Nurse Educ Today.

[10] Grandell-Niemi H, Hupli M, Leino-Kilpi H, Puukka P (2005). Finnish nurses’ and nursing students’ pharmacological skills. J Clin Nurs.

[11] Bertsche T, Niemann D, Mayer Y, Ingram K, Hoppe-Tichy T, Haefeli WE (2008). Prioritising the prevention of medication handling errors. Pharm World Sci.

[12] Manno MS (2006). Preventing adverse drug events. Nursing.

[13] Wolf ZR, Hicks R, Serembus JF (2006). Characteristics of medication errors made by students during the administration phase: a descriptive study. J Prof Nurs.

[14] Mansoury A, Ahmadvand A, Hadjibabaie M, Karger M, Javadi M, Gholami K (2013). Types and severity of medication errors in Iran; a review of the current literature. Daru J Facul Pharm Theran Univ Med Sci.

[15] Harding L, Petrick T (2008). Nursing student medication errors: a retrospective review. J Nurs Educ.

[16] Simonsen BO, Johansson I, Daehlin GK, Osvik LM, Farup PG (2011). Medication knowledge, certainty, and risk of errors in health care: a cross-sectional study. BMC Health Serv Res.

[17] Goldberg D (1985). Identifying psychiatric illness among general medical patients. Br Med J (Clin Res Ed).

[18] Bayne T, Bindler R (1997). Effectiveness of medication calculation enhancement methods with nurses. J Nurs Staff Dev.

[19] Pallant J: ***SPSS Survival Manual A Step by Step Guide to Data Analysis Using SPSS.*** 4th edition. Birkshire England: Mc Graw-Hill Education; 2010.

[20] Manias E (2009). Pharmacology content in undergraduate nursing programs: is there enough to support nurses in providing safe and effective care?. Int J Nurs Stud.

[21] Meechan R, Mason V, Catling J (2011). The impact of an integrated pharmacology and medicines management curriculum for undergraduate adult nursing students on the acquisition of applied drug/pharmacology knowledge. Nurse Educ Today.

[22] Manias E, Bullock S (2002). The educational preparation of undergraduate nursing students in pharmacology: clinical nurses’ perceptions and experiences of graduate nurses’ medication knowledge. Int J Nurs Stud.

[23] Latter S, Rycroft-Malone J, Yerrell P, Shaw D (2000). Evaluating educational preparation for a health education role in practice: the case of medication education. J Adv Nurs.

[24] Morrison-Griffiths S, Snowden MA, Pirmohamed M (2002). Pre-registration nurse education in pharmacology: is it adequate for the roles that nurses are expected to fulfil?. Nurse Educ Today.

[25] Armitage G, Knapman H (2003). Adverse events in drug administration: a literature review. J Nurs Manag.

[26] Schwertz DW, Piano MR, Kleinpell R, Johnson J (1997). Teaching pharmacology to advanced practice nursing students: issues and strategies. AACN Clin Issues.

[27] Tissot E, Cornette C, Limat S, Mourand JL, Becker M, Etievent JP, Dupond JL, Jacquet M, Woronoff-Lemsi MC (2003). Observational study of potential risk factors of medication administration errors. Pharm World Sci.

[28] Whitehair L, Provost S, Hurley J (2014). Identification of prescribing errors by pre-registration student nurses: a cross-sectional observational study utilizing a prescription medication quiz. Nurs Educ Today.

[29] Kelly S, Courts N (2007). The professional self-concept of new graduate nurses. Nurse Educ Pract.

[30] Johnson SA, Johnson LJ (2002). The 4 Cs: a model for teaching dosage calculation. Nurse Educ.

[31] Wright K (2009). The assessment and development of drug calculation skills in nurse education–a critical debate. Nurse Educ Today.

[32] Wright K (2007). Student nurses need more than maths to improve their drug calculating skills. Nurse Educ Today.

[33] Grandell-Niemi H, Hupli M, Leino-Kilpi H (2001). Medication calculation skills of graduating nursing students in Finland. Adv Health Sci Educ Theory Pract.

[34] Grandell-Niemi H, Hupli M, Puukka P, Leino-Kilpi H (2006). Finnish nurses’ and nursing students’ mathematical skills. Nurse Educ Today.

[35] Coyne E, Needham J, Rands H (2013). Enhancing student nurses’ medication calculation knowledge; integrating theoretical knowledge into practice. Nurse Educ Today.

[36] O’Shea E (1999). Factors contributing to medication errors: a literature review. J Clin Nurs.

[37] Wright K (2008). Can effective teaching and learning strategies help student nurses to retain drug calculation skills?. Nurse Educ Today.

[38] Killam LA, Luhanga F, Bakker D (2011). Characteristics of unsafe undergraduate nursing students in clinical practice: an integrative literature review. J Nurs Educ.

[39] Newton JM, McKenna L (2007). The transitional journey through the graduate year: a focus group study. Int J Nurs Stud.

[40] Reid-Searl K, Moxham L, Walker S, Happell B (2010). “Whatever it takes”: nursing students’ experiences of administering medication in the clinical setting. Qual Health Res.

[41] Banning M (2003). Pharmacology education: a theoretical framework of applied pharmacology and therapeutics. Nurse Educ Today.

[42] The Ministry of Education and Research: **National curriculum for nursing education.** Oslo, Norway, URL [https://lovdata.no/dokument/SF/forskrift/2008-01-25-128]

